# Management of Borderline ovarian tumors (BOT): results of a retrospective, single center study in Switzerland

**DOI:** 10.1186/s13048-023-01107-3

**Published:** 2023-01-23

**Authors:** B. Kipp, A. Vidal, D. Lenick, C. Christmann-Schmid

**Affiliations:** Department for Gynecologic Oncology, Cantonal Hospital of Lucerne, Lucerne, Switzerland

## Abstract

**Background:**

Borderline tumors are malignant epithelial ovarian tumors with a very low incidence. Thus experience in diagnostics and treatment is still rare. The aim of this study was to present and analyze data of women with borderline ovarian tumor (BOT) regarding clinical features, histological characteristics, diagnostics and treatment management.

**Methods:**

In this single center retrospective study women with BOT treated at the Departement of Gynecology and Obstetrics at the Kantonsspital Luzern between 2011 and 2018 were analyzed according to their clinical and histological reports.

**Results:**

A total of 42 women were enrolled. The median age was 58.5 with a range from 26 to 85, of which 31 (73.8%) were postmenopausal. Regarding the histological subtypes, 23 women (54.8%) had serous and 15 (35.7%) had mucinous BOT. Seromucinous histology was found in 3 patients (7.1%) and endometrioid in 1 woman (2.4%), respectively. All women underwent surgery. In a total of 39 women (92.9%) a complete surgical staging for BOT was performed. In 29 women (69.0%) staging was performed by laparoscopy, 13 (31.0%) underwent laparotomy. The mean follow up was 52 months (range = 16.3–101.4 months). During this period two patients, initially diagnosed in FIGO stage 1, recurred after 21.7 and 44 months, respectively, the second woman died after 53 months because of the BOT.

**Conclusion:**

In the present study women were treated according to the international therapy recommendations and the rate of recurrence was very low. The most common risk factors for relapse are known to be FIGO stage, incomplete staging and peritoneal implants but were not present in our group. Thus further studies are necessary to investigate additional recurrence risks.

## Introduction

Borderline ovarian tumors (BOT) account for approximately 10–20% of all epithelial ovarian cancers. There is still a gap of knowledge regarding the biological behaviour, optimal surgical extent and adjuvant therapy. The incidence is low and ranges between 1,5–2,5 cases per 100,000 in American women and about 4.8 /100.000 new cases in european series [1]. According to the FIGO classification from 1971 BOTs are considered to be tumors of low malignant potential, the latest WHO classification describes them as atypical proliferative tumors [2]. Histologically BOTs are characterized by complex papillary structures, multilayerd epithelium, only mild nuclear atypia and slightly increased mitotic activity but do not exert destructive stromal invasion [3]. Clinically BOT present similarly to other adnexal mass with often typical features according to the IOTA criteria in the transvaginal ultrasound [4]. Serum CA 125 is recommended preoperatively and in the follow-up setting, however not all women with BOT have elevated serum levels pre-operatively [5]. Furthermore the determination of CA 125 can be useful as a diagnostic tool or in the follow-up setting. To date surgical staging procedure according to the FIGO requirements remains the most important treatment modality in Borderline ovarian tumors including bilateral salpingo-oophorectomy, hysterectomy, peritoneal washing, omentectomy, multiple biopsies – and appendectomy in case of mucinous BOT [6]. As one third of the BOTs occur in women under the age of 40, fertility sparing aspects of staging procedures have been established in the past 10 years [6–8]. Prognosis of BOT is much better compared to invasive epithelial ovarian cancer because of lack of destructive stromal invasion [3, 9]. Additionally BOT present more frequently in FIGO stage 1 limited to the ovary [9, 10]. There is no proven benefit for any adjuvant chemo- or radiotherapy to be associated with increased overall survival. Current guidelines do not recommend any adjuvant treatment in women with BOT regardless of FIGO stage [11, 12]. To date the most important known risk factors for relapse are the initial FIGO stage and the presence of peritoneal implants especially invasive implants [10]. The primary aim of this study was to verify if treatment modalities of women with borderline ovarian tumors at our institution followed the common international therapy recommendations. Secondary we focused on the recurrence rate of all women diagnosed with BOT according to treatment procedures and recurrence risk factors within an 8 year timeframe.

## Patients and methods

This retrospective study was conducted at the certified department of gynaecologic oncology (DKG) at our tertiary referral hospital of the Cantonal hospital of Lucerne, Switzerland. Women with BOT were identified through the institutional cancer centre data base. The medical history and clinical data were obtained from the records including demographics including BMI, age, menopausal status, localization, size, CA 125 level, detailed surgical intervention history, adjuvant therapy and follow-up. The histopathologic findings and detailed information were extracted from the institute of Pathology of the Cantonal hospital of Lucerne. The tissue specimen was analyzed by a specialised team for ovarian tumors at the on-site department of Pathology according to the guideline of the World Health organisation (WHO) International classification for ovarian tumors [13]. Tumor staging was recorded according to the FIGO classification system [14]. According to the AGO guidance (Deutsche Leitlinien AGO) the follow up visit was structured as following: Medical history, transvaginal ultrasound. In case of symptoms further imaging was added. CA 125 levels were not routinely taken. Private gynaecologists were contacted in case of missing follow-up data.

### Statistical analysis

All evaluations were performed in an exploratory, descriptive manner. Categorical variables were analyzed by frequency tables displaying number of cases and percentages. Quantitative variables were summarized by descriptive statistics. Statistical analyses were conducted with STATA (Version 16.1 or later, StataCorp, College Sation, Texas, USA).

The study was approved by the ethics committee Nordwest und Zentralschweiz (2020–00084). It was performed in accordance with the priciples of good clinical practice and the Declaration of Helsinki.

## Results

Between January 2011 and December 2018 a total of 42 women were identified with primary diagnosis of histopathological proven BOT at the department of gynaecologic oncology of the Cantonal Hospital of Lucerne and included in this retrospective study. Demographic characteristics are summarized in Table 1. The median age at the time of diagnosis was 58.5 years (range = 26–85). The majority of women were postmenopausal (73.8%). The body mass index was determined for all women showing a median BMI at the time of diagnosis of 25.5 (range = 18–39). Half of the women had normal weight (50.0%), compared to 11 patients (26.2%) with overweight and 10 patients (23.8%) with obesity. The median follow up was 52 months with a range from 16.3 to 101.4 months. Table 2 summarizes the clinical features, FIGO stage, localisation, CA 125 level and the histological subtypes. Serous borderline tumor (sBOT) was the most prevalent subtype (23/42; 54.8%). Mucinous histology was seen in one third of the cases (15/ 42 35.7%). Only 1 woman had an endometrioid BOT. One mucinous borderline tumor showed microinvasion whereas micropapillary pattern and intraepithelial carcinoma were absent in our collective.

**Table 1:** Demographics

Characteristics	***N*** = 42
**Age**	
Median age (range)	58.5 (26–85)
*Age groups (n (%))*	
< 50 years	12 (28.6%)
≥ 50 years	30 (71.4%)
**Menopause (n (%))**	
No	11 (26.2%)
Yes	31 (73.8%)
**Body mass index (kg/m2)**	
Median BMI (range)	25.5 (18–39)
*BMI groups (n (%))*	
Underweight (< 18)	0 (0.0%)
Normal (18–24.9)	21 (50.0%)
Overweight (25–29)	11 (26.2%)
Obesity (> 29)	10 (23.8%)

**Table 2:** Tumor characteristics by histological subtypes of borderline ovarian tumors

Tumor characteristics		Histological subtype				Overall
		Serous	Mucinous	Seromucinous	Endometroid	
		*N* = 23 (54.8%)	*N* = 15 (35.7%)	*N* = 3 (7.1%)	*N* = 1 (2.4%)	*N* = 42
**FIGO**	I	18 (78.3%)	15 (100.0%)	2 (66.7%)	1 (100.0%)	36 (85.7%)
	II	2 (8.7%)	0 (0.0%)	1 (33.3%)	0 (0.0%)	3 (7.1%)
	III	3 (13.0%)	0 (0.0%)	0 (0.0%)	0 (0.0%)	3 (7.1%)
**Location**	Unilateral	17 (73.9%)	15 (100.0%)	2 (66.7%)	0 (0.0%)	34 (81.0%)
	Bilateral	6 (26.1%)	0 (0.0%)	1 (33.3%)	1 (100.0%)	8 (19.0%)
**CA125 [IU/ml]**	≤ 35	10 (43.5%)	8 (53.3%)	1 (33.3%)	0 (0.0%)	19 (45.2%)
	> 35	10 (43.5%)	6 (40.0%)	1 (33.3%)	0 (0.0%)	17 (40.5%)
	missing	3 (13.0%)	1 (6.7%)	1 (33.3%)	1 (100.0%)	6 (14.3%)
**Microinvasion**		NA	1 (6.7%)	NA	NA	NA

All women with a mucinous BOT had a unilateral presentation whilst one third (26.1%) with a sBOT were bilateral. Most women were diagnosed at FIGO stage I (85.8%). Only 3 women (7.1%) were diagnosed in FIGO stage II and 3 women (7.1%) in FIGO Stage III, respectively. The assessment of CA125 (reference> 35 IU/l) was performed in 36/ 42 (85.7%) women*.* Elevated CA 12–5 level were only detected in one third to 40% of all women included in this study. Regardless of the histopathological findings there was no correlation of the elevated CA 125 levels and FIGO stage, respectively. Table 3 demonstrates the different surgical approaches, interventions and outcome. In 31.0% of women with unclear pre-operative clinical and diagnostic features for BOT or high grade ovarian cancer laparotomy was performed. Although recommended differently by the panel two women only received a cystectomy (1sBOT/ 1 endometrioid BOT). Peritoneal excision and omentectomy were refused by 2 and 3 women respectively. After extensive counselling 4 women (9.6.%) received fertility sparing surgery: ovarian preservation in two women and the other two women with cyst excision only. Complete staging surgery was refused in two women - one of them with bilateral BOT. No pelvic nor a paraaortic lymphnode dissection was performed in any woman. Adjuvant chemotherapy was neither recommended nor performed. During this follow up period 2 women (4.8%) showed a recurrence of disease with one of the two had lethal outcome. A 32-year old women showed recurrence of a mucinous BOT 44 months after primary surgery. Initially she received a laparoscopic cystectomy for diagnostics purpose. Having proven a mucinous borderline ovarian tumor, a fertility-sparing staging procedure including peritoneal biopsies, peritoneal washing, right-sided salpingo-oophorectomy, appendectomy and an infracolic omentectomy was performed during second laparoscopy. The histological diagnosis confirmed a mucinous borderline tumor FIGO stage 1. 44 months later the vaginal ultrasound revealed a suspect cystic mass 5 cm of size of the left ovary. CA 125 and CA 199 were elevated to 89 U/l and 153 U/l, respectively. A diagnostic laparoscopy was performed where a low-grade ovarian carcinoma was histologically confirmed. Consecutively she underwent laparotomy with complete tumor debulking surgery. She received additional chemotherapy with carboplatin and paclitaxel. Unfortunately she died 9 months later. The second woman (49 years) was initially submitted for surgery in 2014 in case of a 13 cm ovarian cyst suspicious of BOT. An initial laparoscopic staging included bilateral salpingo-oophorectomy, peritoneal biopsies, cytologic washing and infracolic omentectomy. The affected ovary was safely removed in an extraction bag. The final diagnosis was a mucinous BOT FIGO stage 1C. 21 months after primary laparoscopic staging she presented with a new pelvic mass of 8 cm, suspicious of recurrent disease in the transvaginal ultrasound including hydronephrosis bilaterally. While CA 125 was normal CA 199 was elevated to 245 U/ml. A subsequent laparotomy was performed achieving complete tumor debulking confirming the initial histology. No adjuvant treatment was recommended. She returned for regular follow-up visits. To date she is recurrent free.

**Table 3:** Procedures and outcomes

Parameter		***N*** = 42
**Surgical procedure**	Laparotomy	13 (31.0%)
	Laparoscopy	29 (69.0%)
**Salpingo-oophorectomy**	Yes	40 (95.2%)
	No	2 (4.8%)
**Ovarian preservation**	Yes	2 (4,8%)
	No	40 (95.2%)
**Hysterectomy**	Yes	28 (66.7%)
	No	5 (11.9%)
	with previous hysterectomy	9 (21.4%)
**PE**	Yes	2 (4.8%)
	No	40 (95.2%)
**Cytology**	Yes	42 (100.0%)
	No	0 (0.0%)
**Omentectomy**	Yes	39 (92.9%)
	No	3 (7.1%)
**Appendectomy**	Yes	20 (47.6%)
	No	19 (45.2%)
	with previous appendectomy	3 (7.1%)
**Recurrence**	Yes	2 (4.8%)
	No	40 (95.2%)
**Death**	Yes	1 (2.4%)
	No	41 (97.6%)

## Discussion

This retrospective study assessed and evaluated 42 women with BOT over a follow-up period of 8 years. Only one woman had a lethal outcome after progression of a mBOT to a low grad ovarian cancer. This confirms the generally good prognosis of this entity although borderline ovarian tumors are known to reoccur even after 15–20 years. Generally the risk of recurrence is low as has been reported in current literature [2, 15]. BOT are mostly diagnosed at the age of 28–62 years around 10 years earlier than invasive ovarian cancer as confirmed in our series with a median age of 58.5 years [1, 7, 8, 16, 17]. In contrast studies of Ji et al., Desfeyx et al. and Pirimoglu et al. reported a lower rate of postmenopausal women affected by a BOT [18–20]. The determination of the CA 125 tumor marker plays an important role in BOT diagnostics pre-operatively and in the follow-up setting. In our study serum CA125 was taken in most women (85.7%) preoperatively. Half of the affected women showed increased levels. Wong et.al found CA125 to be elevated in 39% [21] .Ren et.al had even a higher proportion of elevated CA 125-serum levels (62%). Furthermore they could demonstrate a correlation between elevated serum levels of CA 125 and an advanced-stage BOT [16]. This is in contrast to our results as the majority of women (83%) with elevated serum levels of CA 125 were diagnosed in FIGO stage 1. Ayhan et al. focused on the correlation of elevated serum levels of CA 125, CA 199 and CEA in serous and mucinous BOT. Elevated serum level of CA 125 seems to be associated with serous BOT, whereas in mucinous histology CA199 and CEA were significantly higher compared to serous BOT [22]. Our data does not support the results of Ayhan et.al showing an elevated serum level of CA 12.5 in serous, sero-mucinous and mucinous histology in 43.5%, 33.3% and 40.0%, respectively. Concerning early detection of recurrence Ren et al. could not prove CA 125 to be an independent factor for the probability of recurrent disease [16]. More than 96% of BOTs are of serous or mucinous subtypes. Rare types of BOT are endometrioid, clearcell or transitional cell (Brenner) tumors [9, 23, 24]. In our cohort the prevalence of histological subtypes was comparable to the current literature showing 54.8% serous and 35.7% of mucinous BOT. Recent studies show a distribution of the histopathological types accounting 55–65% for serous tumors and 34–45% for mucinous tumors [17, 25, 26]. In 10–40% they occur bilaterally matching our study [27]. We found one rare entity in a young asian women presenting with a bilateral borderline tumor of endometrioid type. This case was published by our study group 2020 [27]. To date there is no clear statement regarding the significance and associated risk of recurrence in BOT with microinvasion. Hogg et al. and Morris et al. [28, 29] demonstrated no correlation between microinvasion and overall prognosis. In contrast Ren et al. [16] showed a recurrence rate of 39% in BOT showing microinvasion compared to 10% in BOTs without microinvasive architecture. In our study cohort only one woman with a mucinous BOT and microinvasion presented with initially FIGO stage 1. No recurrent disease after 5 years of follow-up. Standard guidelines for surgical treatment recommend a complete staging procedure including bilateral salpingo-oophorectomy, hysterectomy, multiple peritoneal biopsies, omentectomy and peritoneal washing with cytology [11]. For mucinous tumors appendectomy may also be added [6]. Generally surgical approach might be performed by laparoscopy as well as by laparotomy. The retrospective multicentre ROBOT study revealed that recurrence rate and overall survival were not affected by the surgical approach [3]. Cyst rupture (33.9% vs 12.4%) and incomplete staging was significantly more frequent in the laparoscopy group [30]. Associated higher re-operation and recurrence rate must therefore be discussed. As one third of Borderline ovarian tumors occur under the age of fourty fertiliy sparing procedures have been lately established. Available data reveal a higher recurrence rate after conservative treatment including ovarian preservation of at least one ovary (10–20%) compared to radical surgery (5%) without resulting in a higher mortality rate [3, 31–35]. Therefore conservative management of at least part of one ovary and the uterus preservation can be safely offered to young women desiring preservation of fertility. This option must be discussed preoperatively. In this study most women (92.8%) underwent complete surgical staging procedures according to current therapy guidelines. Four women received fertility sparing surgery after informed consent about the fourfold increased risk of recurrence. Trillsch et al. showed in their study that incomplete staging is a negative prognostic factor specially focused on serous borderline tumors with an increased risk of recurrence. Furthermore they demonstrated the highest prognostic impact for omentectomy in unadjusted as well as in multivariate analysis [36]. This data is in contrast to our results where two women with complete staging procedure relapsed. Additionally both women with recurrent disease were initially diagnosed at an early stage (FIGO stage I).

Despite lymph node involvement is described in up to 29% recurrence or survival rate remains similar for women with affected or not affected lymph nodes [19, 37, 38]. Consequently a complete lymphadenectomy is no longer recommended in the current German S3 Guideline and can be omitted as part of the staging procedure [11] Our approach is in line with this recommendation as lymphadenectomy was neither recommended nor performed.

## Conclusion

In conclusion women in this present study were treated according to current guidelines and only two women recurred. In our group neither advanced FIGO stage nor incomplete staging nor peritoneal implants were associated with higher recurrence rate. Further studies are needed to identify additional predictive factors for recurrent disease.

## Data Availability

All data generated or analyzed during this study are included in this published article.
